# Contraction assessment of abdominal muscles using automated segmentation designed for wearable ultrasound applications

**DOI:** 10.1007/s11548-024-03204-0

**Published:** 2024-06-12

**Authors:** Hannah Strohm, Sven Rothluebbers, Luis Perotti, Oskar Stamm, Marc Fournelle, Juergen Jenne, Matthias Guenther

**Affiliations:** 1 Fraunhofer Institute for Digital Medicine MEVIS, Bremen, Germany; 2 mediri GmbH, Heidelberg, Germany; 3 Geriatrics and Medical Gerontology, Charité-Universitaetsmedizin Berlin, Berlin, Germany; 4 Fraunhofer Institute for Biomedical Engineering IBMT, Sulzbach, Germany; 5 University of Bremen, Bremen, Germany

**Keywords:** Deep learning, Optical flow, Real-time systems, Segmentation, Rehabilitative ultrasound imaging

## Abstract

**Purpose:**

Wearable ultrasound devices can be used to continuously monitor muscle activity. One possible application is to provide real-time feedback during physiotherapy, to show a patient whether an exercise is performed correctly. Algorithms which automatically analyze the data can be of importance to overcome the need for manual assessment and annotations and speed up evaluations especially when considering real-time video sequences. They even could be used to present feedback in an understandable manner to patients in a home-use scenario. The following work investigates three deep learning based segmentation approaches for abdominal muscles in ultrasound videos during a segmental stabilizing exercise. The segmentations are used to automatically classify the contraction state of the muscles.

**Methods:**

The first approach employs a simple 2D network, while the remaining two integrate the time information from the videos either via additional tracking or directly into the network architecture. The contraction state is determined by comparing measures such as muscle thickness and center of mass between rest and exercise. A retrospective analysis is conducted but also a real-time scenario is simulated, where classification is performed during exercise.

**Results:**

Using the proposed segmentation algorithms, 71% of the muscle states are classified correctly in the retrospective analysis in comparison to 90% accuracy with manual reference segmentation. For the real-time approach the majority of given feedback during exercise is correct when the retrospective analysis had come to the correct result, too.

**Conclusion:**

Both retrospective and real-time analysis prove to be feasible. While no substantial differences between the algorithms were observed regarding classification, the networks incorporating the time information showed temporally more consistent segmentations. Limitations of the approaches as well as reasons for failing cases in segmentation, classification and real-time assessment are discussed and requirements regarding image quality and hardware design are derived.

## Introduction

For patients with chronic low back pain, exercise therapy has shown to be as effective as conservative treatments in reducing pain level [1]. During therapy, the abdominal drawing-in maneuver is an often used segmental stabilizing exercise aiming at the contraction of the musculus (M.) transversus abdominis (TrA) without parallel activation of the M. obliquus externus (OE) and M. obliquus internus (OI). Because learning the isolated contraction of the TrA is often challenging for patients, it can be guided by a physiotherapist either by manual palpation but also by using ultrasound imaging. The latter is feasible since muscle activation has shown to be accompanied by a change in muscle thickness [2] which can be detected via ultrasound imaging [3]. Comparing TrA thickness during rest and exercise has shown a significant increase when the muscle is contracted [4]. Besides acting as a diagnostic tool, the ultrasound video can additionally be used as direct biofeedback for the patient. Studies show that such feedback can shorten the time patients need to learn the correct execution of the exercise [5]. To monitor the correct execution, thickness values of the muscles can be derived from ultrasound videos which is often done offline and manually [3, 4]. Therein, the muscle thickness can either be measured at fixed anatomical positions or by manually segmenting the muscle and averaging along several measurement positions. As the annotation effort grows with the sequence length, manual measures are often restricted to selected frames. Automated approaches to measure muscle thickness can be based on ultrasound video tracking algorithms [6]. A recent approach [7] used deep learning to locate edgepoints on the TrA contour which are intended to be used for thickness measurements. A more general approach to the segmentation of cross-sectional areas of different muscles is described in [8] where different state-of-the-art deep learning algorithms are compared.

The following work investigates three strategies to automatically segment the three abdominal muscles in ultrasound videos showing an abdominal drawing-in maneuver. Resulting segmentations are used to automate thickness measurements which indicate whether muscles are contracted by comparing between rest and exercise. The work is embedded in a recent research project ULTRAWEAR^1^ which aims to develop an ultrasound wearable that monitors the execution of segmental stabilizing exercises, both at physiotherapy and during home training. Especially the home-use scenario requires a software solution that automatically provides feedback to the exercising patient. Such algorithms can also eliminate the need for manual measures and speed up offline evaluation of exercises, independent of the type of ultrasound scanner used. Of the segmentation approaches investigated, two are deep learning based while the third approach combines a deep neural network (DNN) with optical flow tracking. From the automated segmentations, muscle thickness and center of mass were calculated to assess the contraction status of each muscle. Thresholds for both measures to separate contraction from non-contraction were defined based on manual reference segmentations. A retrospective analysis was performed but also a real-time approach was evaluated.

## Material and methods

### Data

Video data from eleven volunteers (mean age = $41\pm 13$, BMI = $25\pm 4.2$, ten male, one female) performing an abdominal drawing-in maneuver were recorded. Each video consists of an initial resting phase of ${10\,\mathrm{\text {s}}}$, followed by ${10\,\mathrm{\text {s}}}$ of exercise and a second rest of ${4\,\mathrm{\text {s}}}$. Volunteers performed between two and four exercise executions in supine position with ${90\,\mathrm{{}^{\circ }}}$ to ${100\,\mathrm{{}^{\circ }}}$ knee flexion and ${40\,\mathrm{{}^{\circ }}}$ to ${60\,\mathrm{{}^{\circ }}}$ hip flexion following verbal instructions. The ultrasound transducer was positioned at navel level on the left side and adjusted to include the transition to the lumbodorsal fascia of the TrA. For data acquisition, the 256 channel version of the multi-channel electronics DiPhAS (Fraunhofer IBMT, St. Ingbert, Germany) was used for driving a ${4.6\,\mathrm{\text {M}\text {Hz}}}$ linear probe with 192 elements. As the presented work aims to be utilized in a mobile application setting where only portable ultrasound electronics with fewer channels are available, we tried to replicate this scenario. Therefore, the 192 element aperture has been divided into 96 transmit only (odd) and 96 receive only (even) elements. The transmit elements have been further divided in groups of 4 elements and the same transmit delay has been applied to all elements in one group. On receive side, only the elements with even numbers have been used during the reconstruction.

Data were manually annotated by two research associates of the Geriatrics Research Group at Charité Berlin and a student assistant. For each frame in the video sequences, the abdominal muscles were segmented, and the contraction state (relaxed or contracted) was classified. Sequences with insufficient image quality, for example caused by non-optimal transducer positioning, were discarded, resulting in 26 of 33 available videos being manually annotated. Annotated sequences were distributed into four groups. Three groups containing 19 videos from eight subjects were used in a cross-validation scheme for training and validation of the developed algorithms. The fourth group with seven videos from three subjects was used solely for testing. During data splitting, it was assured that videos of the same subject are kept in the same group.^2^

### Segmentation algorithms

#### 2D segmentation (2D DNN)

For a 2D approach, MobileNetV3 with a LRASPP head for semantic segmentation [9] was used. Due to highly correlated video data, transfer learning with pretrained weights was employed. Furthermore, only a subselection of ten frames per subject from both resting and exercise state with minimal inter-frame correlation was used. Images were normalized to fit the pretrained network weights and downsampled by a factor of two to increase the receptive field of the network. Data augmentation was applied to increase the number of training samples, including vertical flipping, scaling, altering the brightness and applying Gaussian blurring, as well as optical distortion, grid distortion and elastic transformation [10]. Training was performed in a patch-wise fashion (size [200, 200]) with a batchsize of 5. The learning rate was set to $10^{-4}$ and Adam with a weight decay of 0.01 and cross-entropy loss was used. The final model was selected at the point during training were the validation loss reaches its minimum.

#### 2D segmentation with tracking (2D DNN+OF)

To include the temporal information of the video, a combination of the previously described 2D neural network and optical flow tracking (2D DNN+OF) was implemented. The first frame of each video was segmented using the 2D DNN, and this initial segmentation was propagated along the video using an OF algorithm. For the latter, the iterative Lucas–Kanade method [11] was used which calculates a flow field between the current frame and a reference frame for a set of sparse features. Using interpolation, motion vectors for every pixel can be derived. The following parameters for the OF algorithm were experimentally determined: $\textrm{ofSigma} = 0.04$, $\mathrm {fgs\_lambda} = 5000$, $\mathrm {fgs\_sigma} = 3$. The reference frame for tracking was determined by selecting the frame with highest correlation from the set of already segmented frames which must not necessarily be the direct predecessor of the current frame. This strategy helped recover the correct muscle shapes after transition from the exercise state back into rest. Every fifth frame was considered to serve as a reference frame to limit the effect of propagating mistakes. Propagated masks were smoothed with a Gaussian filter with kernelsize fifteen.

#### U-Net-LSTM

To include temporal information from the video directly into a deep learning network, a U-Net [12] with convolutional long short term memory (convLSTM) layers [13] was designed. It consists of an encoder and a decoder with three levels where each level comprises two convolutional layers. In the encoder, the first convolution was replaced with a bidirectional convLSTM. The U-Net used dropout with rate 0.2 and instance normalization after the convolutional layers. As input to the network, subseries of 16 frames were extracted from the videos with a mutual distance of 16 frames. Those subseries were constructed starting from every seventh frame to get a representative selection from each video. Similar to the 2D DNN, images were downsampled and their value range was shifted to [0,1]. The same data augmentation techniques and training hyperparameter were used. Due to memory limitations, training was only possible with a batchsize of one, therefore gradient accumulation with stepsize five was used. During inference, sequences up to 16 frames can be provided to the U-Net-LSTM. From the 16th frame one, several predictions for one frame are available since each prediction is derived from a different subseries of input frames. From all candidate predictions, those having the most predecessor frames were selected. For inference on the test data, model results from the cross-validation training were combined using majority voting. Only the largest predicted object of each muscle was kept while considering the segmentation of the previous frame to enforce consistency over time. Holes in the masks were closed assuming the muscle to be a uniform surface.

### Contraction state assessment

Two features were analyzed to automatically classify the contraction status of a muscle based on its segmentation: the thickness of the muscle and its center of mass. Both measures were determined for each muscle and timepoint individually. The thickness was obtained by averaging vertical measurements along the segmentation masks at four defined positions with respect to the left image border. From the center of mass coordinates, only the lateral one was kept for further analysis as the left–right movement of the muscle can be considered a meaningful indicator of contraction.

For the retrospective analysis, thickness and lateral center of mass measurements were grouped according to their belonging to either the resting or the exercise state and overlaps $\delta _{\textrm{th}}$ and $\delta _{\textrm{com}}$ of their distributions were calculated. During contraction, the thickness or center of mass is assumed to change significantly resulting in a decreased overlap between distributions. In general, to retrieve the overlap between two distributions *A* and *B*, the value $\hat{\alpha }$ is sought such that

1 $$\begin{aligned} \hat{\alpha } = \arg \min _{\alpha } P_{\alpha }^{A} >= P_{100-\alpha }^{B}, \end{aligned}$$

where $P_{\alpha }$ is the $\alpha \mathrm {-th}$ percentile of the distribution. Assuming that the values from *B* are generally greater compared to *A*, their overlap is defined as

2 $$\begin{aligned} \delta = 100-\hat{\alpha }. \end{aligned}$$

**Fig. 1:**
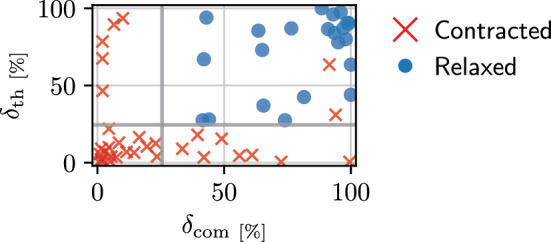
Validation data: overlaps between rest and exercise distributions for thickness ($\delta _{\textrm{th}}$) and center of mass ($\delta _{\textrm{com}}$) measured on manual segmentation masks. Each point represents one individual muscle from the 19 available videos. Thresholds to separate contraction states are determined as $t_{\textrm{th}}=24.5$ and $t_{\textrm{com}}=25.5$

**Fig. 2:**
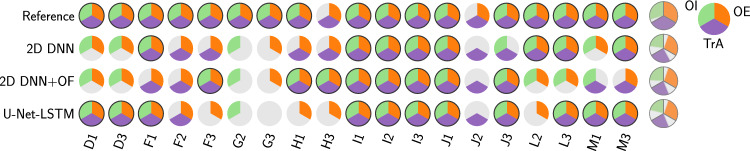
Validation data: classification results based on automated thickness and lateral center of mass measurements performed on segmentation masks generated by different algorithms (y-axis). The “Reference” row refers to the manual annotation. Each circle represents the three abdominal muscles of one subject. Capital letters refer to the different subjects while the numbers denote different exercise executions. When a muscle is classified correctly, the corresponding third of the circle is colored. Outer right circles represent the overall percentage of correct classifications

Muscle thickness is assumed to increase in case of a contraction. This implies that distribution *A* holds values from the resting and *B* from the exercising phase. In our study, we observed that muscle contraction can be accompanied by a shift to the left; therefore, the center of mass is expected to decrease. For $\delta _{\textrm{com}}$, distribution *A* contains values from the exercise phase, whereas *B* those from the resting phase. Thresholds $t_{\textrm{th}}$ and $t_{\textrm{com}}$ to separate contracted from relaxed muscles based on the distribution overlaps were defined using the manual reference segmentations.

It was observed that for very narrow distributions the calculated overlaps could be small, while at the same time the distribution means are too close together to actually display a change in thickness or center of mass. Such uncertainty can result from the measuring process where a small shift of the transducer could lead to close but narrow distributions. Therefore, the exercise state distributions were shifted using displacements $d_{\textrm{th}}$ and $d_{\textrm{com}}$ which were optimized together with the overlap thresholds.

A real-time scenario where the contraction status is obtained during exercise was simulated. The same approach as described for the retrospective analysis was used to classify the state, but instead of considering all measurements from the exercise, frames were summarized into blocks of approximately one second each. Only frames from the first resting period were considered. Processing times of the different algorithms were measured to validate their real-time capability.

## Results

First, thresholds for the distribution overlaps $\delta _{\textrm{th}}$ and $\delta _{\textrm{com}}$ as well as the displacements $d_{\textrm{th}}$ and $d_{\textrm{com}}$ were determined using the manual segmentations on the cross-validation data. Displacements were optimized via a grid search to be $d_{\textrm{th}} = -1$ and $d_{\textrm{com}}=6$. Overlap values for all 57 muscles (3 muscles per 19 videos each) as determined by Eqs. 1 and 2 are shown in Fig. 1. Crosses and circles indicate contracted and relaxed muscles, respectively. Thresholds were set such that a maximum number of correctly classified muscles is achieved while maximizing the distance between the samples and both thresholds, resulting in $t_{\textrm{th}} = 24.5$ and $t_{\textrm{com}} = 25.5$. Muscles with overlap values below one threshold were classified to be contracted. It can be observed that using only the thickness measure, seven muscles out of 57 would be classified incorrectly. Combining both criteria, this can be reduced to two.

**Fig. 3:**
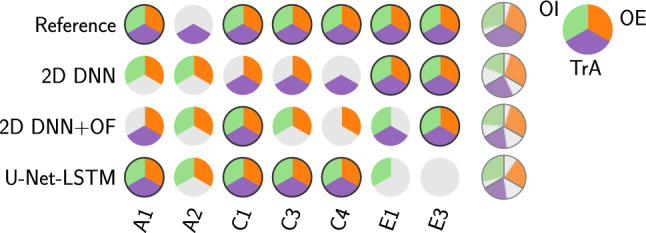
Test data: classification results based on segmentation masks generated by different algorithms similar to Fig. 2

**Fig. 4:**
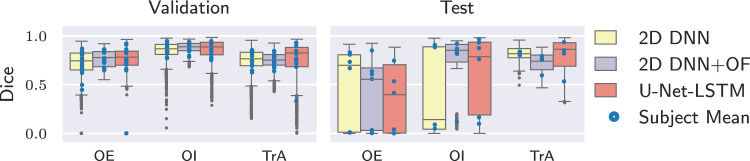
Dice coefficient for the three segmentation algorithms for retrospectively correctly classified muscles. The mean of each subject is displayed (circles) to visualize potential outliers on subject level

The defined thresholds were applied to distributions derived from segmentation masks generated by the three algorithms described in the “Segmentation algorithms” section. Results for the retrospective analysis for subjects from the cross-validation folds are shown in Fig. 2. Each circle represents one subject with its three abdominal muscles. Capital letters (D to M) refer to different subjects while the numbers label different exercise executions. When the classification matches the reference annotation the corresponding third is colored. Using the manual reference segmentation, 55 of 57 (96.5%) muscles are classified correctly and for 17 of 19 subjects all three muscles are correct. From the segmentation algorithms, both the 2D DNN approach and its combination with OF classify 43 muscles correctly (75.4%) and are completely correct for eight subjects. The U-Net-LSTM reaches 41 correct muscles (72%) and classifies 11 subjects correctly. Similarly, results for the test set are depicted in Fig. 3. With the manual segmentation 19 muscles are classified correctly (90.5%) and six from seven subjects. All automated algorithms classified 15 muscles correctly (71.4%). While both the 2D DNN and the 2D DNN with OF are correct for two subjects, the U-Net-LSTM is correct for four subjects.

**Fig. 5:**
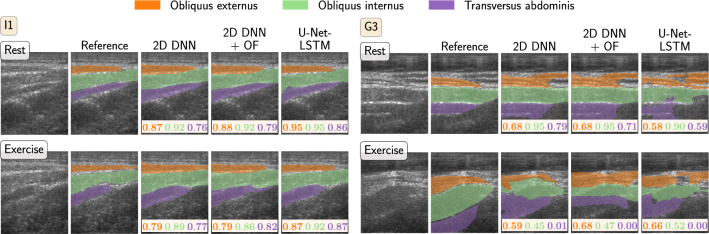
Segmentation results of the three algorithms for videos from two subjects. For each subject an exemplary frame from the resting and exercise state is shown. Left side: subject I1, which is correctly classified by all algorithms. Right side: subject G3 which is mostly misclassified using the automated segmentation algorithms

**Fig. 6:**
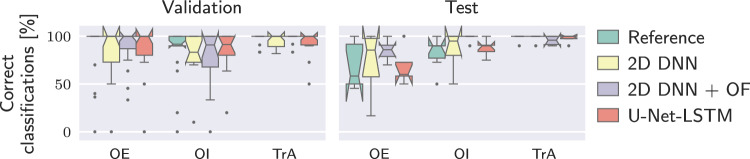
Percentage of correct classifications for a real-time approach making an assessment every second including only muscles which are retrospectively classified correctly

To assess the quality of the segmentations obtained by the different algorithms independently from their ability to provide the correct classification result, the Dice coefficient was computed. It relates the overlap of the reference mask R and the automatically generated mask Y to their combined areas:

3 $$\begin{aligned} \text {Dice} = \frac{2 |R \cap Y|}{|R + Y|}. \end{aligned}$$

An overview of the Dice coefficient considering only retrospectively correctly classified muscles is shown in Fig. 4. In addition to its distribution, the mean Dice of every individual subject is plotted as circle. For the validation data the median Dice values between the segmentation algorithms are similar. In particular, outliers with low Dice scores can be observed for the 2D DNN. Distributions of Dice values for the test data appear broader especially for the 2D DNN and U-Net-LSTM when considering OE and OI. The mean of the individual subjects are distributed more broadly indicating that the segmentation quality differs more between subjects than between frames of the same video.

Exemplary segmentations from all three algorithms for videos of two subjects are displayed in Fig. 5. For both subjects a frame from the resting and exercise state was selected to visualize the differences which can occur between those states. For subject I1, all algorithms came to the correct classification result (Fig. 2). According to the reference annotation, the TrA is contracted during the exercise while OE and OI are relaxed. This is reflected in the thickening of the TrA as well as in a shift to the left which is visible in the manual segmentation. Even though the automatic segmentations do not perfectly align with the reference, all capture the thickness increase of the TrA and also partly the shift. For the second subject shown (G3), the algorithms mostly fail to correctly classify the contraction status. In the reference annotation, all three muscles are contracted during exercise, visible in both an increase in thickness as well as movement of the muscles. Here, the automatic algorithms especially have problems to delineate the muscles during exercise.

**Table 1:** Runtime measurements for the segmentation algorithms and the thickness and center of mass measures. Values are averaged over 800 runs

	Mask computation	Thickness and center of mass	Total
2D DNN	${16.3\,\mathrm{\text {m}\text {s}}} \pm {12.9\,\mathrm{\text {m}\text {s}}}$		${17.6\,\mathrm{\text {m}\text {s}}} \pm {16.8\,\mathrm{\text {m}\text {s}}}$
2D DNN+OF	${37.9\,\mathrm{\text {m}\text {s}}} \pm {12.2\,\mathrm{\text {m}\text {s}}}$	${1.3\,\mathrm{\text {m}\text {s}}} \pm {3.9\,\mathrm{\text {m}\text {s}}}$	${39.2\,\mathrm{\text {m}\text {s}}} \pm {20.7\,\mathrm{\text {m}\text {s}}}$
U-Net-LSTM	${219.6\,\mathrm{\text {m}\text {s}}} \pm {13.8\,\mathrm{\text {m}\text {s}}}$		${220.9\,\mathrm{\text {m}\text {s}}} \pm {17.7\,\mathrm{\text {m}\text {s}}}$

For real-time analysis, the same threshold and displacement values as for the retrospective approach were used. The percentage of correct assessments during exercise for muscles classified correctly in the retrospective analysis is shown in Fig. 6. Considering the validation data, the median of the correct classifications is above 80% for all algorithms and for the OE and TrA around 100%. Outliers appear especially for the OE and the OI where in some cases even none of the assessments are correct. For the test data, the correctly classified updates for the OI and TrA show a distribution similar to the validation data. For the OE, the percentages of correct classifications are lower. Compared to the validation data, there are less extreme outliers. Time measurements for the segmentation algorithms as well as for computing thickness and center of mass are listed in Table 1. Values are averaged over 800 runs of the algorithms on different images from the cross-validation set. The neural networks were executed on a Tesla T4. The 2D DNN can be executed fastest, followed by its combination with optical flow. The U-Net-LSTM needs considerably more time to segment a single frame. Computation time for thickness and center of mass is the same for all algorithms and is negligible. Computing the overlaps between the measurement distributions to classify into contracted or relaxed additionally needs ${57.22\,\mathrm{\text {m}\text {s}}} \pm {21.83\,\mathrm{\text {m}\text {s}}}$. However, this operation has to be executed only once per assessment, which means every second.

**Fig. 7:**
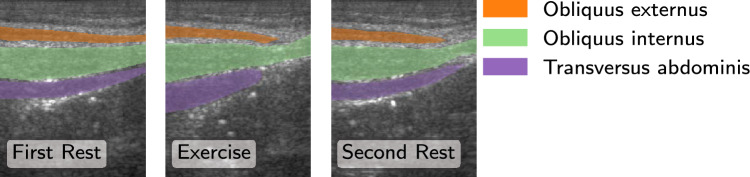
Subject F3: Exemplary frames from rest and exercise with manual annotations. During exercise only the TrA contracts. The visible left-shift of the OE could be interpreted as contraction when comparing to the first rest only as it is done in a real-time application

## Discussion

Ultrasound wearables provide large amounts of data which deep learning can help to analyze and make accessible to the user. The purpose of this work is to provide a basis for real-time analysis of ultrasound video data by comparing different algorithms for segmenting abdominal muscles. The segmentations were utilized for contraction assessment during a segmental stabilizing exercise in physiotherapy.

Although muscle thickness measurements are commonly used to assess muscle contraction, they are often done manually. To the best of our knowledge, there is no standardization for automated thickness measurement. For data in this study, it has been shown that automatic measures based on manual segmentations are not always sufficient to separate between contracted and relaxed muscles (Fig. 1). Possible reasons are that the part of the muscle which thickens is outside the captured region or that the contraction results in minimal thickening which cannot be resolved. Including the center of mass to account for muscle shifts during contraction, classification accuracy increases. Similar to the thickness, it was observed that the center of mass alone would not be sufficient as discriminator and that both criteria do not have to be necessarily fulfilled for a contracted muscle. Evaluation on the test data indicates that the optimized thresholds are applicable to new data.

Performance of the three segmentation algorithms can be evaluated on muscle level but evaluation in terms of number of correctly classified subjects is also important. An automatic system can only provide feedback regarding the exercise, when all three muscles are classified correctly. Out of the three investigated segmentation algorithms, the 2D DNN and 2D DNN+OF performed best regarding the overall number of correctly classified muscles. However, in terms of the number of correctly classified subjects, the U-Net-LSTM performs best on both training (11/19) and test data (4/7).

For some cases, all algorithms had difficulties in providing the correct classification result (Fig. 5). For G3, the muscles do not only greatly increase in thickness, but also move such that the TrA partially leaves the imaging region. In combination with decreased visibility of the lower fascia, this can be an explanation for the poor segmentation performance. Especially for the 2D DNN which does not use information of the previous frames, segmentation is challenging.

Although the classification results in this study rely on segmentations, it can be observed that correct classification is possible using imperfect segmentations. For I1 (Fig. 5), the manual segmentation does not include the fascia as part of the muscle, whereas the automatic segmentations do. Yet, classification results are correct, indicating that the overall changes in muscle thickness and position are captured. Looking at the overall segmentation quality (Fig. 4), several outliers with low Dice scores can be observed especially for the 2D DNN. Nevertheless, the classification result can be correct, as long as the insufficient segmentations only make up a small part. The 2D DNN+OF obtain slightly higher Dice coefficients with fewer outliers indicating a higher temporal consistency of the segmentations. This could be of advantage in cases where the segmentation mask itself is of importance, e.g., for computation of quantitative thickness measures. The wider spread of Dice values for the test data can be attributed to two videos from subject A where a fat layer above the muscles is incorrectly classified as part of the OE. Since such an anatomy was not represented in the training data, the networks fail to correctly differentiate between fat layer and muscle.

For real-time analysis on all retrospectively correct classified muscles, it was observed that classification is correct for the majority of assessments. However, some muscles are misclassified throughout the whole exercise (outliers in Fig. 6). Partially this can be attributed to field of view shifts, as exemplary shown for F3 in Fig. 7. In the reference annotation, only the TrA is contracted during exercise, but the OE shifts to the left which can be interpreted as contraction when comparing to the first rest. Considering the second rest, it appears likely that a displacement of the transduce caused the shift. As ultrasound data in this study was acquired with a handheld device, such displacements cannot be ruled out. For a wearable device, this brings up the requirement of a robust, flexible attachment which allows exercise movements without field of view shift. Regarding processing times, the 2D DNN and its combination with OF can be executed fast enough to be able to process each frame, whereas inference time of the U-Net-LSTM is too long to be applicable in real time with the given framerate. The delay of about ${57.22\,\mathrm{\text {m}\text {s}}}$ to classify the muscles is considered fast enough for sending real-time feedback to the user.

The cohort in this study consists of mainly male volunteers without a known history of back pain. However, the application scenario envisioned with the feedback system is to support elderly patients with low back pain. As muscles of elderly people are likely to differ in their appearance, a next step would be to extent the cohort with our target group. Also, more data from female subjects should be included. Data acquired with the specifically designed wearable device should also be included as DNN solutions tend to have difficulties in being transferred to different devices.

## Conclusion

The benefit of ultrasound wearables depends not only on the hardware and imaging quality, but especially for home-use scenarios also on the algorithm used to evaluate the acquired data. Designing a feedback system for supporting segmental stabilizing exercises, we compared three different deep learning approaches which segment the abdominal muscles and assess their contraction state via automated measurements. For the independent test set, 71% of muscles were classified correctly by the algorithms whereas with manual reference segmentations, an accuracy of 90% was reached. While we did not observe a substantial difference regarding the number of correctly classified muscles, the U-Net-LSTM performs best in terms of correctly classified subjects. The two approaches incorporating the time information showed temporally more consistent segmentations. Besides retrospective evaluation also real-time application was shown to be principally feasible. The latter comes with a slight decrease in classification performance due to a decreased number of frames which can be used for averaging. We observed that reduced visibility of the muscle fascia led to imperfect segmentations and incorrect classifications. Here, an improvement in image quality is expected to further improve segmentation and at the same time classification accuracy.

## Footnotes

1 ULTRAsound based WEARable as biofeedback-system for effective training in chronic back pain.

2 Group distribution: Fold 1: M1, M3, G2, G3, H1, H3; Fold 2: D1, D3, I1, I2, I3, J1, J2, J3; Fold 3: F1, F2, F3, L2, L3; Test: A1, A2, E1, E3, C1, C3, C4. Videos with the same leading character belong to the same subject.
